# Thyroid metastasis in a patient with hepatocellular carcinoma: case report and review of literature

**DOI:** 10.1186/1477-7819-5-144

**Published:** 2007-12-24

**Authors:** Hung-Hua Liang, Chih-Hsiung Wu, Ka-Wai Tam, Chiah-Yang Chai, Sey-En Lin, Soul-Chin Chen

**Affiliations:** 1 Department of Surgery, Taipei Medical University Hospital, Taipei, Taiwan; 2 Department of Pathology, Taipei Medical University Hospital, Taipei, Taiwan

## Abstract

**Background:**

Despite the apparent low incidence of cancer metastatic to the thyroid, autopsy and clinical series suggest it is more common than generally. Although lung, renal, and breast cancer are probably the most common primary sites, a number of cancers have been reported to metastasize to the thyroid synchronously with diagnosis of primary tumor or years after apparently curative treatment.

**Case presentation:**

We report a rare case of a hepatocellular carcinoma metasatic to the thyroid. The patient presented seven months after original diagnosis and treatment with hepatic lobectomy with multiple neck lesions producing a mass effect on the trachea and bilateral lymphadenopathy. Fine-needle aspiration revealed highly anaplastic carcinoma, and immunohistochemistry confirmed hepatocellular carcinoma. The patient received total thyroidectomy as palliative therapy because of the presence of multiple recurrent lesions in the liver.

**Conclusion:**

Clinicians should consider the possibility of metastatic cancer in each patient who presents with a new thyroid mass, especially those with a history of cancer, however remote. In cases where cytology or histology is not diagnostic, immunohistochemistry may be definitive in making the diagnosis.

## Background

Although metastatic disease in the thyroid is infrequently seen, both autopsy and clinical series indicate the problem is more common than generally thought. Autopsy series on patients with cancer have yielded incidence rates ranging from 1.25% to as high as 24%, and some authors have suggested that the incidence of metastatic disease in the thyroid may have risen during the twentieth century [1,2]. Recent reports suggest that the most common primary sites are the kidney, lung, breast, and gastrointestinal tract [1-7]. However, a wide variety of cancers may metastasize to the thyroid, including nasopharyngeal carcinoma, choriocarcinoma, osteosarcoma [1], leiyomyosarcoma, liposarcoma [5], melanoma [3], and tumors of neuroendocrine origin [6,8].

Our clinical experience with a patient whose hepatocellular carcinoma (HCC) metastasized to the thyroid led to a review of the literature, where three other cases involving HCC were identified [9,10]. Discussion of these cases highlights possible difficulties in evaluating these patients, ranging from making the diagnosis to evaluating thyroid disease in the context of the patient's condition in order to make treatment decisions and determine prognosis. Our experience in Asia allows us to see differences in the clinical profile of thyroid metastasis compared with that in Western countries; this may be useful to physicians who see patients from various parts of the world.

## Case presentation

A 54-year-old man was admitted for weight loss, upper abdominal distension with a palpable mass, and epigastric pain for one month. Dull pain had worsened and the abdominal mass had become increasingly evident for the preceding week. His history was remarkable for Hepatitis B infection for more than 20 years. Physical examination was negative for spider angioma, shifting dullness, jaundice or scleral icterus, hand flapping tremor, or thyroid mass. Laboratory data were normal: serum aspartate aminotransferase, 18 IU/L; alanine aminotransferase, 18 IU/L; creatinine, 0.8 mg/dL. Abdominal ultrasonography revealed a mixed echoic mass at level S3–4 (maximum diameter, 9.8 cm), and the entire liver parenchyma had heterogeneous echogenicity. Computed tomography (CT) of the liver revealed multiple foci of heterogeneously enhanced mass lesions. The largest was roughly 8.9 cm × 6.7 cm. Serum alpha-fetoprotein was elevated with a level of 162.4 ng/mL (normal, < 12 ng/ml).

Left hepatic lobectomy was performed two weeks after initial diagnosis, and histopathologic analysis revealed a moderately to poorly differentiated HCC. The cut surface revealed a well-defined, tan-white, firm tumor measuring 8.4 cm × 6.5 cm × 5.5 cm with obvious necrosis. The tumor directly invaded the liver capsule and was adherent to fat on the anterior superior surface. The surgical margin was 1 cm from the tumor and microscopically negative for tumor cells. Intraoperatively, two well-defined, black spongy tumors measuring up to 2 cm in diameter were noted near the main tumor mass. No liver cirrhosis, cholestasis, or portal vein thrombosis was seen.

Microscopically, the tumor featured cells growing in solid nests and a pseudoglandular, as well as a focal trabecular pattern. Individual cells showed marked nuclear pleomorphism and prominent nucleoli with abundant eosinophilic granular cytoplasm and frequent mitotic figures. There was extensive fibrosis and necrosis. Perineural invasion was found near the hilum, but no invasion of blood or lymphatic vessels was evident. Mucicarmine stain was negative for mucin in the carcinoma. In scattered areas, cells were immunochemically positive for cytokeratin (CK) 7 and negative for CK 20. The two small black and spongy tumors were typical of cavernous hemangioma. No liver cirrhosis was noted. After complete pathologic examination, the patient's pTNM stage according to the AJCC Cancer Staging Manual (6^th ^Edition) [11] was pT1N0M0 (solitary tumor without vascular invasion).

**Figure 1:**
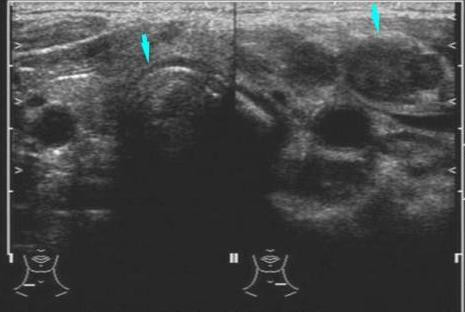
Computed tomography of the neck revealed the left thyroid tumor (arrow).

At follow-up two months after surgery, no local recurrence or metachronous HCC was observed. The patient had a normal CT and alpha-fetoprotein level had markedly decreased to 66.5 ng/mL. Four months after surgery, abdominal CT revealed multiple poorly defined hypodense lesions (largest about 1 cm) in segments VII and VIII. Alpha-fetoprotein level was 119.7 ng/mL. Transarterial embolization therapy was performed for recurrent disease. Seven months after surgery alpha-fetoprotein level was 102.9 ng/mL and recurrent HCC was found. Transarterial embolization therapy was performed again. At this time the patient reported new-onset dyspnea and several neck nodules. Physical examination revealed a firm, hard left thyroid mass with bilateral lymphadenopathy. Ultrasonography revealed multiple nodules in the left thyroid with heterogeneous echogenicity (Figure 1). Fine needle aspiration of the largest thyroid nodule was performed, and cytology revealed poorly differentiated carcinoma.

**Figure 2:**
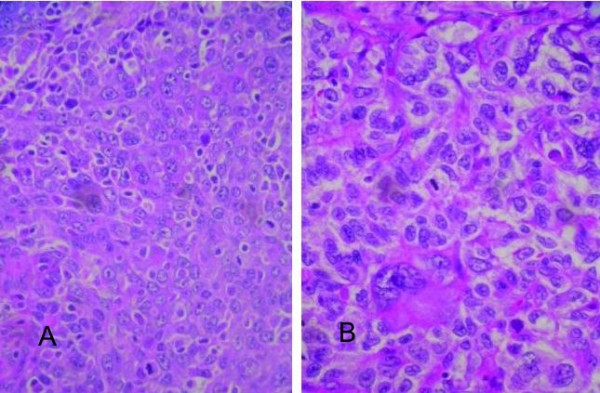
**A**. Metastatic hepatocellular carcinoma (HCC) in the thyroid gland (hematoxylin-eosin, 400×). **B**. Primary HCC (hematoxylin-eosin, 400×).

**Figure 3:**
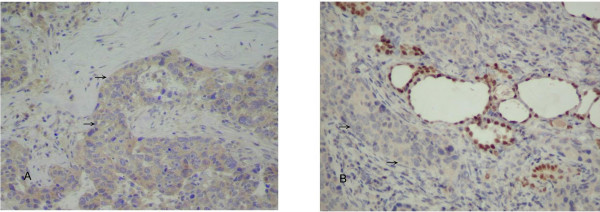
**A**. Immunohistochemical staining of the metastatic carcinoma was positive for alpha-fetoprotein (arrow). **B**. Negative control staining picture.

Immunohistochemical staining showed that the thyroid carcinoma was negative for thyroid transcription factor-1 (TTF-1), CK 7, CK 20, and thyroglobulin. Because the carcinoma was similar microscopically to the HCC found in the liver (Figure 2A, B) and positive for alpha-fetoprotein (Figure 3A, B), a tentative diagnosis of metastatic HCC with bilateral lymph node involvement was made. Eight months after the original hepatic lobectomy, the patient underwent total thyroidectomy with bilateral regional lymph node dissection. Both dysphagia and dyspnea resolved postoperatively. The patient expired eight months after the thyroidectomy due to recurrent HCC complicated by sepsis.

## Discussion

Metastatic thyroid cancer may be more common than primary thyroid malignancy, especially in patients who have a history of cancer [7]. A variety of presentations, from neck masses felt by the patient to nodular goiters discovered on physical examination have been reported, as have lesions revealed on imaging studies [1,2,7,9,12]. Our patient's presentation with multiple nodules, bilateral lymphadenopathy, and dysphagia and dyspnea, strongly suggested advanced local or metastatic cancer. In light of the patient's recurrent HCC and results of fine needle aspiration of the thyroid lesion, both the surgeon and pathologist believed it more likely the thyroid mass represented metastatic HCC than a primary neoplasm. Total thyroidectomy with regional lymph node dissection was performed and pathological examination confirmed the diagnosis of metastatic HCC. A previous case report of HCC metastatic to the thyroid indicates that a markedly different clinical picture can develop with the same primary neoplasm. In this case, an elderly man had no history of cancer presented with a single neck mass that moved with swallowing, which could well have been primary thyroid cancer [9].

Hematogenous and lymphogenous pathways for metastasis to the thyroid have been suggested [13]. Although one would consider hematogenous metastasis via a major vein would be most likely with HCC, it is possible that our patient had lymphogenous metastasis, with disease spreading to one or more nodes before spreading to the thyroid gland itself. Only a series of cases with careful imaging and pathologic evaluation will determine which route of metastasis is more common with HCC.

Fine needle aspiration is often used to obtain tissue for analysis; however, it may be difficult to distinguish primary from metastatic disease in instances when cells are highly anaplastic [2]. In the case reported by Masuda *et al *[9], fine needle aspirate cytology was not diagnostic, and a core needle biopsy was obtained. The tissue showed histological architecture resembling a hepatocellular cord and positive immunostaining for alpha-fetoprotein. The primary tumor was discovered on abdominal imaging.

The use of immunohistochemistry has allowed far more undifferentiated cancers to be correctly identified than was possible in 1960, when Elliott and Frantz published their paper on metastatic carcinomas 'masquerading' as primary thyroid cancers [3]. Because only 20% to 30% of anaplastic thyroid carcinomas are positive for thyroid markers such as thyroglobulin, it is important to be aware of any previous malignancies so appropriate marker staining can be done [2]. In our case, the thyroid sample was negative for TTF-1, CK 7, and CK 20, but positive for alpha-fetoprotein, which is relatively specific for HCC, although of low sensitivity [14]. A report on use of immunohistochemistry to differentiate several malignancies, including HCC, that are difficult to distinguish by histology and cytology noted that negative stain results for anaplastic cytology may not be informative in identifying the site of origin, and biopsy should be considered [14].

We concur with the recommendation made by several previous authors that all patients with a history of cancer, however remote, should be evaluated for possible metastasis when a new thyroid lesion is discovered [2,7]. In our review of the literature, metastases appeared 15 years [1,7], 16 years [6], 24 years [2], and 42 years [10] after the diagnosis of the primary cancer (the last two cases were renal cell carcinoma and colorectal carcinoma primaries, respectively). We also agree with Lam and Lo [4], who compared cases of Chinese and Japanese patients with those from the West and concluded that differences in primary sites between Western and Eastern countries probably reflect incidence rates of cancers in a region rather than different biological behavior.

## Conclusion

Knowledge of an individual patient's personal and family history, as well as region of origin, may be very useful in determining what immunostains to perform for patients with unknown primary tumor and highly anaplastic thyroid metastasis. Patients with widely disseminated cancer have poor prognoses, but patients with a single thyroid metastasis may have improved quality of life and longer survival if the correct diagnosis is made and treated accordingly.

## Abbreviations

CK, cytokeratin; 

CT, computed tomography; 

HCC, hepatocellular carcinoma; 

TTF-1, thyroid transcription facter-1.

## Competing interests

The author(s) declare that they have no competing interests.

## Authors' contributions

HHL performed literature review, drafted and revised manuscript. KWT Evaluated radiological features and contributed to radiological part of manuscript, CYC Carried out initial assessment of the patient and helped in draft of manuscript. SCC Contributed to the concept of the report. CHW Operated on the patient and corrected the manuscript for it scientific content. SEL Evaluated histopathological features and contributed histological part. All authors read and approved the final manuscript.
